# Evaluating the Influence of a Psychiatry Shadowing Programme on the Clinical Readiness of New Psychiatry Residents at Hamad Medical Corporation: A Comparative Cross-Sectional Study

**DOI:** 10.1192/bjo.2026.11349

**Published:** 2026-06-30

**Authors:** Sazgar Hamad, Ahmad Alater, Zidan Masoudi, Mugtaba Osman, Majid Alabdulla

**Affiliations:** 1 Hamad Medical Corporation, Doha, Qatar; 2 Naufer Center, Doha, Qatar

## Abstract

**Aims:**

To assess whether a structured psychiatry shadowing programme at Hamad Medical Corporation (HMC) is associated with improved clinical preparedness among trainees and to examine whether motivation and well-being modify the relationship between shadowing quality and preparedness.

**Methods:**

In 2024, a comparative cross-sectional anonymous online survey was distributed to psychiatry trainees at HMC (N=45). Participants were grouped by completion of the shadowing programme. Preparedness was measured across core clinical domains including psychiatric interviewing, management of psychiatric emergencies, independent patient management, electronic medical record use, and multidisciplinary team collaboration. Perceived skills gained, satisfaction, and attitudes toward the programme were captured. Multiple linear regression assessed predictors of overall preparedness (shadowing quality, motivation, well-being). Moderation analyses examined whether motivation and well-being modified the association between shadowing quality and preparedness.

**Results:**

Of 45 respondents, 26 (57.8%) had completed the shadowing programme. Confidence in conducting psychiatric interviews increased significantly, with the proportion rating themselves as “confident” rising from 4.4% before shadowing to 28.9% after shadowing (χ²=15.556; p=0.016). The most frequently reported gains were familiarity with the electronic medical record system (48.9%) and psychiatric interviewing skills (46.7%). Shadowing quality was a strong predictor of preparedness (β=0.46; p<0.0001). Motivation and well-being were not independent predictors but significantly moderated this relationship (well-being: p=0.0012; motivation: p=0.030), indicating stronger benefits of high-quality shadowing among trainees with higher well-being and motivation. No adverse outcomes were reported.

**Conclusion:**

As one of the first evaluations of a structured shadowing programme in a psychiatry residency context, this study demonstrates a meaningful association between high-quality shadowing and trainee clinical readiness. Findings support the integration of structured shadowing into early residency curricula, alongside parallel investment in trainee well-being and engagement to maximise educational impact.

